# Pooled safety analysis of pimitespib for the treatment of patients with advanced gastrointestinal stromal tumors

**DOI:** 10.1007/s10147-025-02917-9

**Published:** 2025-11-20

**Authors:** Yukinori Kurokawa, Noboru Yamamoto, Yuko Hirano, Naoki Arimura, Chunlan Sun, Toshihiko Doi

**Affiliations:** 1 Department of Gastroenterological Surgery, The University of Osaka Graduate School of Medicine, Osaka, Japan; 2 Department of Experimental Therapeutics, National Cancer Center Hospital, Tokyo, Japan; 3 Taiho Pharmaceutical Co., Ltd., Tokyo, Japan; 4 Department of Experimental Therapeutics, National Cancer Center Hospital East, 6-5-1 Kashiwanoha, Kashiwa-Shi, Chiba, 277-8577 Japan

**Keywords:** Gastrointestinal stromal tumors, Gastrointestinal toxicity, Heat shock protein 90 (HSP90) inhibitor, Ocular toxicity, Pimitespib, Safety

## Abstract

**Background:**

Pimitespib is a first-in-class heat shock protein 90 (HSP90) inhibitor, approved in Japan for the treatment of advanced gastrointestinal stromal tumors (GIST). This study aimed to characterize the incidence and time course of adverse drug reactions (ADRs) associated with pimitespib.

**Methods:**

This was a post hoc analysis of pooled safety data from a phase 1 (NCT02965885), phase 2 (jRCT2080223127), and phase 3 (jRCT2080224033) study of pimitespib in patients with advanced GIST. The present analysis included Japanese study participants who received oral pimitespib 160 mg once daily for 5 days followed by 2 days’ rest per week in 21-day cycles. Pooled safety outcomes included the incidence and severity of ADRs; ADRs leading to treatment modifications or discontinuation; and the time to ADR onset and resolution.

**Results:**

In total, 119 patients were included. ADRs were reported in 114 patients (95.8%); gastrointestinal ADRs were most common (99 patients [83.2%]; most often diarrhea [75.6%]) and ocular ADRs occurred in 26 patients (21.8%; most often night blindness [11.8%]). Median time to first onset of any gastrointestinal ADR was 3.0 days; the outcome of gastrointestinal ADRs was recovered/recovering in 61 patients (61.6%), with a median time to resolution of 44.0 days. Median time to first onset of any ocular ADR was 19.0 days; ocular ADRs were recovered/recovering in 20 patients (76.9%), with a median time to resolution of 21.0 days.

**Conclusions:**

This analysis suggests that most ADRs associated with pimitespib are manageable and reversible, thus supporting its use in patients with advanced GIST.

## Introduction

Gastrointestinal stromal tumors (GIST) refer to a heterogeneous group of soft tissue sarcomas in the gastrointestinal tract [1]. Most GIST derive from the precursors of the interstitial cells of Cajal, and the majority (~ 80–90%) first present in the stomach or small intestine. Mutations in the genes encoding the receptor tyrosine kinase protein (*KIT*) and platelet-derived growth factor receptor-α (*PDGFRA*) account for approximately 60–70% and 10–15% of GIST, respectively [1].

Increased understanding of the molecular features of GIST has given rise to targeted therapies that have improved the prognosis for patients with advanced or metastatic disease. In particular, *KIT* and *PDGFRA* mutations in GIST cause the activation of receptor tyrosine kinases that promote cell proliferation and survival via the major mitogen-activated protein kinase (MAPK) and PI3K/AKT/mTOR signaling pathways [1]. Tyrosine kinase inhibitors (TKIs; e.g., imatinib, sunitinib, regorafenib, ripretinib) were hypothesized to block the constitutive activation of KIT- and PDGFRA-mediated pathways, and have indeed demonstrated anti-tumor activity in clinical trials of patients with advanced GIST [2–8]. Based on these findings, imatinib is now the standard first-line treatment option for patients with advanced or metastatic GIST, while sunitinib and regorafenib are the recommended second- and third-line options, respectively [9, 10]. Ripretinib is additionally recommended as a fourth-line TKI therapy in European clinical practice guidelines [9], but is not currently approved in Japan.

Although the availability of TKIs have provided important treatment options for patients with advanced GIST, subsequent-line therapies of the same drug class can present some clinical challenges. In addition to acquired resistance mechanisms that can impact the effectiveness of subsequent-line TKI therapy (reviewed elsewhere [1, 11]), TKIs also share a common adverse event (AE) profile that can impact treatment continuity in some patients. Due to both on- and off-target effects, TKIs are associated with a wide range of cardiovascular, gastrointestinal, hepatic, dermatological, and other AEs [12], which can impair treatment adherence [13, 14], and therefore a patient’s ability to continue current- and subsequent-line TKI therapy. In order to optimize survival outcomes for patients with advanced GIST, treatment options beyond TKIs are needed.

Pimitespib (TAS-116) is a first-in-class heat shock protein 90 (HSP90) inhibitor in development for the treatment of GIST and other solid tumors [15, 16]. The HSP90 family are molecular chaperones that regulate the stability of several proteins involved in tumor development (including KIT and PDGFRA); therefore, inhibition of HSP90 with pimitespib may represent an alternative treatment strategy to TKIs [15, 16]. The safety and efficacy of pimitespib in advanced GIST have been evaluated in a series of phase 1, 2, and 3 studies, which consistently demonstrated its tolerability and anti-tumor activity in patients who were refractory to previous TKI therapy [17–19]. In June 2022, pimitespib was first approved in Japan for the treatment of GIST that has progressed after standard TKI therapies [15, 20], and is now the recommended fourth-line treatment for advanced GIST in Japanese clinical practice guidelines [10].

Given its novel mechanism of action, pimitespib has a safety profile that is distinct from standard TKI therapy. In particular, HSP90 inhibitors have been associated with gastrointestinal and ocular AEs in previous cancer studies [21–25]; therefore, it is important to characterize pimitespib-related AEs so they can be effectively managed in clinical practice. However, due to the limited number of patients in each clinical study, the extent of the data from each individual clinical trial on the time to onset and resolution of AEs was limited. Herein we present a pooled analysis of safety data from the phase 1, 2, and 3 pimitespib trials, in order to more comprehensively understand the incidence and time course of adverse drug reactions (ADRs) in patients with advanced GIST.

## Patients and methods

### Study design

This was a pooled post hoc analysis of data from three clinical studies that evaluated the safety of pimitespib in patients with advanced GIST [17–19]. Full methods for each study are described in their respective trial publications; methods relevant to the present analysis are summarized in the following subsections.

#### Phase 1 study

As previously described, a phase 1 study was conducted to elucidate the maximum tolerated dose (MTD), safety, pharmacokinetics, and efficacy of pimitespib in patients with advanced solid tumors (NCT02965885, jRCT2080222394) [17]. Briefly, the study was conducted in the United Kingdom and Japan, and included 61 adults with advanced solid tumors that were no longer responsive to standard treatment. All enrolled patients with advanced GIST in this study were Japanese.

The study comprised two phases; in both phases, pimitespib was administered orally under fasted conditions, in 21-day cycles. During the initial dose escalation phase, patients assigned to step 1 received pimitespib once daily (QD) at a dose ranging from 4.8 to 150.5 mg/m^2^, to determine the MTD for QD dosing. Patients assigned to step 2 received pimitespib once every other day (QOD) at a dose ranging from 107.5 to 295.0 mg/m^2^, to determine the QOD MTD. During the subsequent expansion phase, patients assigned to step 1 received pimitespib 160 mg QD for 5 consecutive days followed by 2 days’ rest per week, while patients assigned to step 2 received pimitespib 340 mg QOD.

#### Phase 2 study

A single-arm, phase 2 study was conducted in Japan to evaluate the safety and efficacy of pimitespib for the treatment of advanced GIST (jRCT2080223127) [18]. The study included 40 patients with metastatic or unresectable GIST that was refractory to imatinib, sunitinib, and regorafenib.

All patients received pimitespib 160 mg, administered orally under fasted conditions, QD for 5 consecutive days followed by 2 days’ rest per week in 21-day cycles.

#### Phase 3 study

The methods and primary results of the phase 3 CHAPTER-GIST-301 study have previously been published (jRCT2080224033) [19]. Briefly, CHAPTER-GIST-301 evaluated the efficacy and safety of pimitespib as fourth-line therapy in patients with advanced GIST in Japan. The study included 86 patients with advanced GIST who progressed on or were intolerant to prior imatinib, sunitinib, and regorafenib.

During an initial double-blind treatment period, patients were randomized 2:1 to receive pimitespib (*n* = 58) or matched placebo (*n* = 28). Pimitespib 160 mg was administered orally, QD for 5 consecutive days followed by 2 days’ rest per week in 21-day cycles. Patients remained in the double-blind treatment period until progressive disease (PD) was confirmed by blinded central radiological review, at which point patients could switch to (or continue) receiving pimitespib during an optional open-label treatment period. At the time of the primary analysis, 1 patient in the pimitespib arm and 17 in the placebo arm had PD during the double-blind period and crossed over to open-label pimitespib treatment.

### Pooled safety outcomes

In all three studies, the safety of pimitespib was assessed through the incidence of AEs, which were graded according to the National Cancer Institute Common Terminology Criteria for Adverse Events (CTCAE version 4.03) [17–19]. In particular, ocular toxicities were monitored with regular ophthalmological examinations.

The present analysis included Japanese patients with advanced GIST who received pimitespib 160 mg QD for 5 consecutive days followed by 2 days’ rest per week in either of the phase 1, 2, or 3 studies. The primary safety outcome was the incidence of ADRs, which were defined as AEs for which there was a reasonable possibility of a causal relationship with pimitespib, as determined by the study investigators. A reasonable possibility of a causal relationship with pimitespib was defined across all 3 studies, as: (1) those that occurred at a time considered to be related to the administration of pimitespib; (2) could not be adequately explained by the primary disease, complications, or concomitant medications; (3) demonstrated a clinical course consistent with pimitespib as the cause (e.g., recovery or improvement of the adverse event) upon discontinuation of pimitespib, or (4) showed pharmacologically or clinically certain reproducibility when re-administered as necessary.

AEs and ADRs were coded using system organ class and preferred terms of the Medical Dictionary for Regulatory Activities (MedDRA) version 23.0, and event severity was graded using CTCAE version 4.03. Additional safety outcomes included the incidence of serious ADRs; ADRs leading to treatment interruption, dose reduction, or treatment discontinuation; the time to onset and resolution of major ADRs; and gastrointestinal and ocular ADRs of clinical interest.

### Statistical analysis

Baseline characteristics and safety outcomes for the pooled analysis population are summarized using descriptive statistics, including numbers and proportions for categorical variables, and medians and ranges for continuous variables. Statistical analyses were performed using SAS software, version 9.4 (SAS Institute, Cary, NC, USA).

## Results

### Study population

In total, 119 Japanese patients with advanced GIST received pimitespib 160 mg QD for 5 consecutive days followed by 2 days’ rest per week in the phase 1, 2, or 3 studies, and were subsequently included in this pooled safety analysis. This included 4 patients with advanced GIST from the phase 1 study, all 40 patients from the phase 2 study, and 75 patients from the phase 3 study (i.e., 58 patients who were randomized to pimitespib, plus 17 patients who crossed over from placebo to pimitespib during the open-label treatment period).

The median age of the pooled safety analysis population was 62.0 years and 58.8% were male (Table 1). All had received at least three prior systemic therapies before pimitespib.

**Table 1:** Baseline patient characteristics (pooled safety analysis population)

	*N* = 119
Male sex, n (%)	70 (58.8)
Age (years), median (range)	62.0 (26–83)
< 65, n (%)	75 (63.0)
≥ 65, n (%)	44 (37.0)
ECOG PS, n (%)	
0	97 (81.5)
1	22 (18.5)
Site of primary tumor, n (%)^a^	
Small intestine	65 (56.5)
Stomach	39 (33.9)
Other	11 (9.6)
Number of prior systemic therapies, n (%)	
3	71 (59.7)
4	32 (26.9)
≥ 5	16 (13.4)

^a^Data based on *n* = 115; site of primary tumor not available for patients from the phase 1 study (*n* = 4)

ECOG PS, Eastern Cooperative Oncology Group Performance Status

### Pooled safety outcomes

**Fig. 1:**
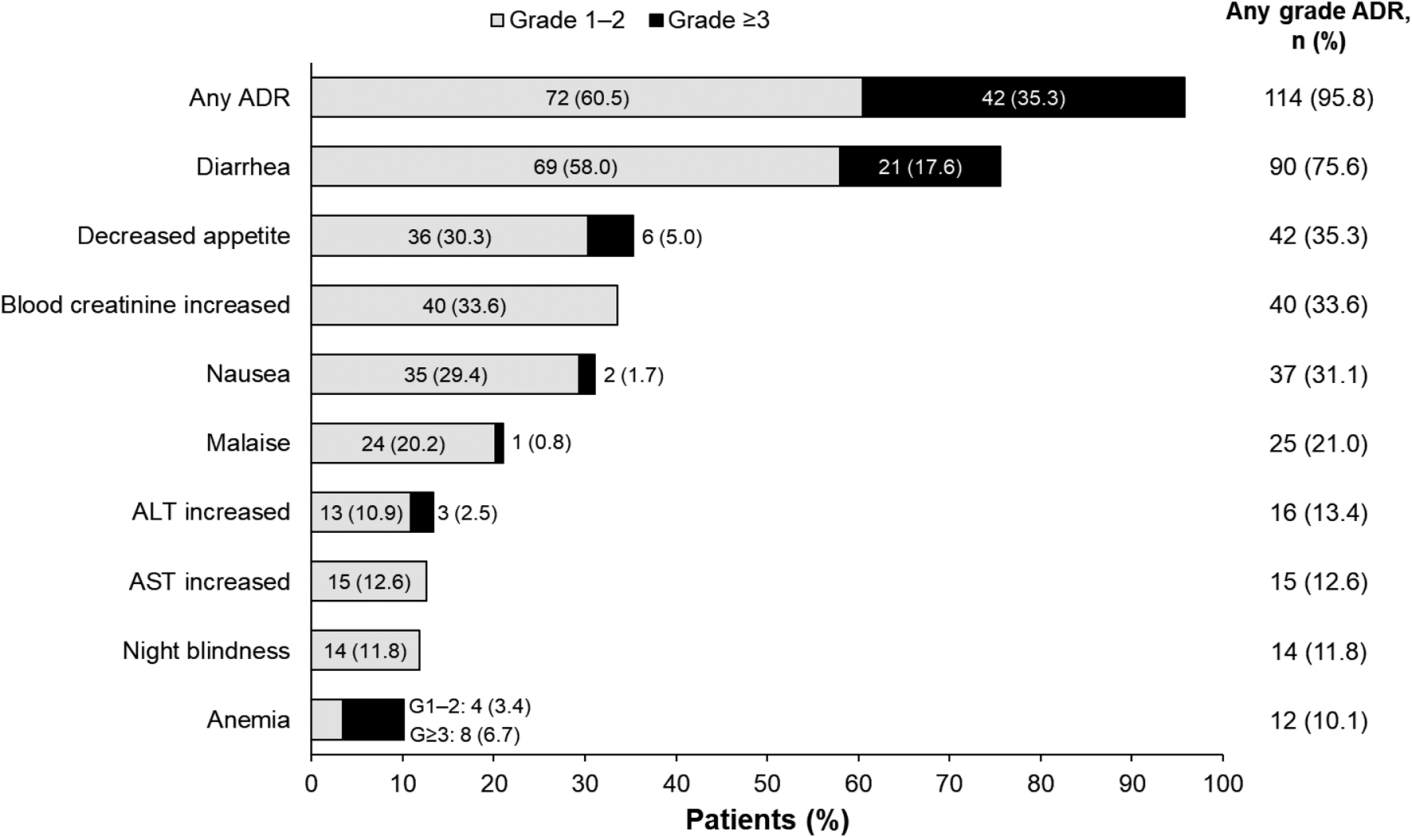
Incidence of common adverse drug reactions (pooled safety analysis population). Common ADRs were defined as those that occurred in ≥ 10% of patients in the pooled safety analysis population. For patients with multiple occurrences of the same ADR, the highest-grade ADR is counted only once. ADR, adverse drug reaction; ALT, alanine aminotransferase; AST, aspartate aminotransferase; G, grade

Overall, ADRs of any grade were reported in 114 patients (95.8%) included in the pooled safety analysis population (Fig. 1). The most common ADR was diarrhea (75.6%), followed by decreased appetite (35.3%), increased blood creatinine (33.6%), and nausea (31.1%). ADRs of grade ≥ 3 severity occurred in 42 patients (35.3%).

Serious ADRs were reported in 14 patients (11.8%). Serious ADRs that occurred in > 1 patient were decreased appetite (4 patients [3.4%]) and diarrhea (3 patients [2.5%]); those that occurred in 1 patient each (0.8%) were anemia, febrile neutropenia, duodenal ulcer hemorrhage, enterocolitis, intra-abdominal hemorrhage, abnormal liver function, liver disorder, and dehydration. Three patients (2.5%) had an ADR leading to pimitespib treatment discontinuation, including retinal vein occlusion, liver disorder, and drug eruption in 1 patient each (0.8%). No ADRs led to death among included patients.

The incidence of ADRs leading to pimitespib treatment interruption or dose reduction are summarized in Table 2. Overall, ADRs leading to treatment interruption were reported in 75 patients (63.0%) and ADRs leading to pimitespib dose reduction were reported in 46 patients (38.7%). The most common ADRs were diarrhea (31.1% and 17.6%, respectively). The median relative dose intensity was 79.4% (range: 20.0%–100.0%).

**Table 2:** Adverse drug reactions leading to treatment interruption or dose reduction in ≥ 1.0% of patients (pooled safety analysis population)

n (%)	*N* = 119	
	Treatment interruption	Dose reduction
Any ADR	75 (63.0)	46 (38.7)
Gastrointestinal disorders	46 (38.7)	27 (22.7)
Diarrhea	37 (31.1)	21 (17.6)
Nausea	10 (8.4)	8 (6.7)
Vomiting	3 (2.5)	0
Investigations	26 (21.8)	7 (5.9)
Blood creatinine increased	13 (10.9)	4 (3.4)
ALT increased	7 (5.9)	2 (1.7)
AST increased	7 (5.9)	1 (0.8)
Blood alkaline phosphatase increased	4 (3.4)	1 (0.8)
Blood bilirubin increased	2 (1.7)	1 (0.8)
Blood lactate dehydrogenase increased	2 (1.7)	0
Neutrophil count decreased	2 (1.7)	0
Metabolism and nutrition disorders	16 (13.4)	10 (8.4)
Decreased appetite	13 (10.9)	10 (8.4)
Dehydration	3 (2.5)	0
General disorders and administration site conditions	12 (10.1)	7 (5.9)
Malaise	7 (5.9)	7 (5.9)
Pyrexia	3 (2.5)	0
Fatigue	2 (1.7)	0
Blood and lymphatic system disorders	7 (5.9)	2 (1.7)
Anemia	6 (5.0)	2 (1.7)
Infections and infestations	5 (4.2)	1 (0.8)
Cystitis	3 (2.5)	0
Pneumonia	2 (1.7)	1 (0.8)
Eye disorders	5 (4.2)	3 (2.5)
Vision blurred	2 (1.7)	1 (0.8)
Renal and urinary disorders	5 (4.2)	5 (4.2)
Renal impairment	2 (1.7)	3 (2.5)
Hepatobiliary disorders	4 (3.4)	2 (1.7)
Hepatic function abnormal	2 (1.7)	1 (0.8)
Liver disorder	2 (1.7)	1 (0.8)
Nervous system disorders	2 (1.7)	1 (0.8)
Dysgeusia	2 (1.7)	1 (0.8)

ADR, adverse drug reaction; ALT, alanine aminotransferase; AST, aspartate aminotransferase

Multiple occurrences of the same ADR in one patient are counted only once

### Time to onset and resolution of ADRs

**Fig. 2:**
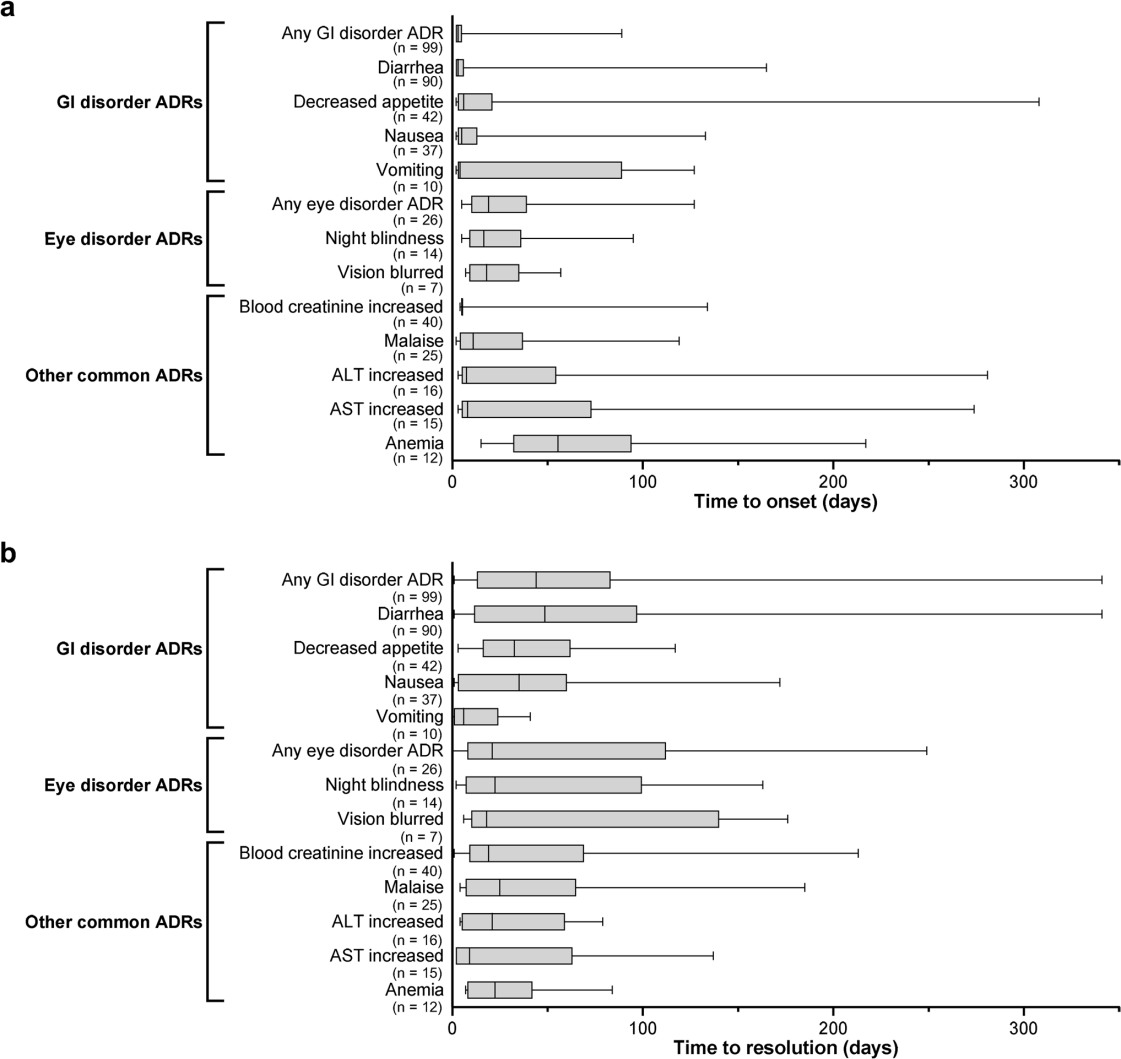
Boxplots of (**a**) time to onset and (**b**) time to resolution for selected adverse drug reactions (pooled safety analysis population). Selected events were GI and eye disorder ADRs that occurred in ≥ 5% of patients in the pooled safety analysis population, and other ADRs that occurred in ≥ 10% of patients in the pooled safety analysis population. ADR, adverse drug reaction; ALT, alanine aminotransferase; AST, aspartate aminotransferase; GI, gastrointestinal, n, number of events

Time to onset and time to resolution analyses for selected ADRs are presented in Fig. 2. The median time to first onset for any gastrointestinal ADR was 3.0 days after the first administration of pimitespib, and the median time to resolution was 44.0 days after first ADR onset. Among the gastrointestinal ADRs that occurred in ≥ 5% of patients, the median time to first onset ranged from 3.0 days for diarrhea to 6.0 days for decreased appetite. Median time to resolution among common gastrointestinal ADRs ranged from 6.0 days for vomiting to 48.5 days for diarrhea. Pimitespib treatment was interrupted for grade 3 or higher gastrointestinal ADRs and could also be interrupted for grade 1 or 2 ADRs at the investigator’s discretion in the phase 3 study [19].

The median time to first onset for any ocular ADR was comparatively longer than for any gastrointestinal ADR (19.0 days), while the median time to resolution was comparatively shorter (21.0 days; Fig. 2). Among the ocular ADRs that occurred in ≥ 5% of patients, the median time to first onset was 16.5 days for night blindness and 18.0 days for blurred vision. Median time to resolution for these common ocular ADRs were 22.5 and 18.0 days, respectively. In the phase 3 study, ocular ADRs were managed by dose interruption of pimitespib for grade 2 or higher events and for Grade 1 events at the investigator’s discretion in accordance with the protocol [19]. In addition, ophthalmologic monitoring was performed at baseline and at the end of treatment; monitoring included interviews, correcting of visual acuity, tonometry, fundoscopy, slit-lamp microscopy, optical coherence tomography, and color vision testing, with additional examinations as needed to evaluate ocular ADRs [19].

### Gastrointestinal and ocular ADRs

**Fig. 3:**
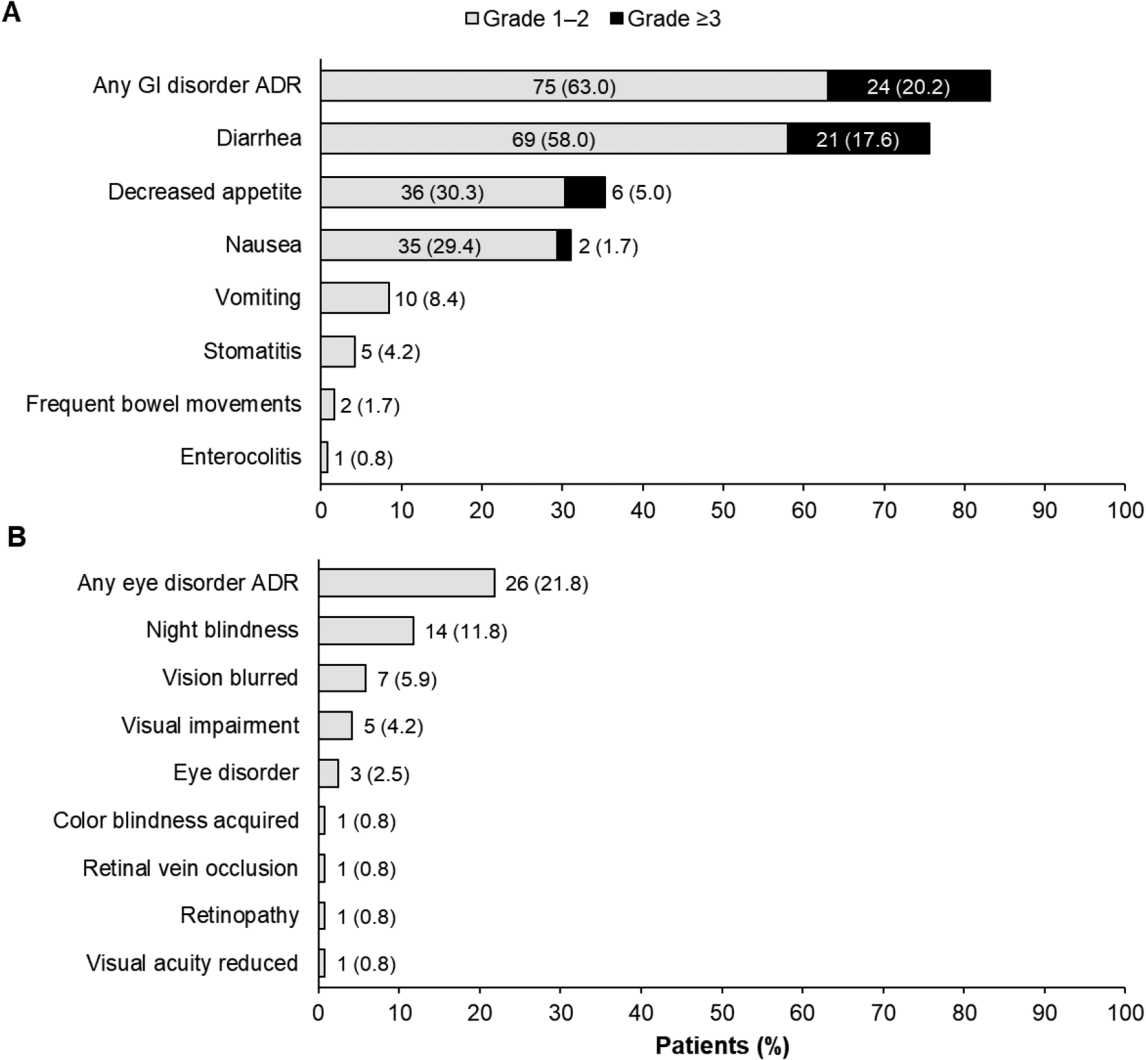
Incidence of (**a**) gastrointestinal and (**b**) ocular adverse drug reactions (pooled safety analysis population). For patients with multiple occurrences of the same ADR, the highest-grade ADR is counted only once. ADR, adverse drug reaction; GI, gastrointestinal

Overall, gastrointestinal ADRs of clinical interest occurred in 99 patients (83.2%), including 24 patients (20.2%) with gastrointestinal ADRs of grade ≥ 3 severity (Fig. 3a).

Among the 99 patients with gastrointestinal ADRs, the outcome of the ADR was recovered/recovering in 61 patients (61.6%), not recovered in 37 patients (37.4%), and unknown in 1 patient (1.0%). The outcome of diarrhea ADRs (the most common gastrointestinal ADR) was recovered/recovering in 68/90 patients (75.6%) and not recovered in 21/90 patients (23.3%).

In total, ocular ADRs of clinical interest were reported in 26 patients (21.8%; Fig. 3b). The most common ocular ADR was night blindness (11.8%), followed by blurred vision (5.9%), visual impairment (4.2%), and eye disorder (2.5%). Ocular ADRs of acquired color blindness, retinal vein occlusion, retinopathy, and reduced visual acuity occurred in 1 patient each (0.8%). The majority of ocular ADRs (92.3%) were grade 1 in severity and no grade ≥ 3 ocular ADRs were observed. As described above, ocular ADRs that led to pimitespib treatment interruption and dose reduction occurred in 5 (4.2%) and 3 (2.5%) patients, respectively (Table 2), while retinal vein occlusion led to treatment discontinuation in 1 patient (0.8%).

Among the 26 patients with ocular ADRs, the outcome of the ADR was recovered/recovering in 20 patients (76.9%) and not recovered in 6 patients (23.1%). The outcome of night blindness ADRs (the most common ocular ADR) was recovered in 12/14 patients (85.7%) and not recovered in 2/14 patients (14.3%).

## Discussion

This pooled safety analysis evaluated the tolerability of pimitespib, a first-in-class HSP90 inhibitor approved in Japan for treatment of patients with advanced GIST. Pooled data found that pimitespib demonstrated an acceptable safety profile, with few serious ADRs or events leading to treatment discontinuation, and no ADRs leading to death. Gastrointestinal and ocular ADRs with pimitespib, that are common to HSP90 inhibitors, were often grade 1–2 in severity, and most were manageable and reversible with pimitespib treatment interruption, dose reduction, and/or supportive therapies. Overall, these safety data support the use of pimitespib for the treatment of advanced GIST in Japan.

Overall, the most common ADRs observed in this pooled analysis—namely gastrointestinal and ocular events, and abnormal blood/laboratory findings—were broadly consistent with previous studies of HSP90 inhibitors in patients with cancer, including advanced GIST [21–25]. However, unlike several previous HSP90 inhibitors that have had clinical development programs halted due to dose-limiting toxicity [26, 27], data from the present analysis demonstrated the overall acceptable safety profile of pimitespib. Although from different drug classes, when comparing the safety profile of pimitespib (HSP90 inhibitor) with another fourth-line treatment option, ripretinib (TKI inhibitor) in Europe, both medicines have shown similar favorable safety profiles [5, 19]

In particular, diarrhea was the most common ADR observed in this analysis, while other common gastrointestinal events included decreased appetite and nausea. The onset of gastrointestinal ADRs after pimitespib initiation typically occurred earlier than other common ADRs, but also tended to be manageable with treatment interruption, dose reduction, and/or supportive therapies. Pimitespib can cause severe diarrhea that may lead to dehydration and serious kidney dysfunction. Therefore, it is important to take appropriate measures, such as administering antidiarrheal medications, providing fluid replacement, and, if necessary, interrupting or reducing the dosage of pimitespib. Additionally, to reduce patient anxiety, it is important to inform them prior to initiating treatment about the possibility of diarrhea, its associated symptoms, and strategies for managing this ADR. Although the outcome of gastrointestinal ADRs was not recovered in approximately 37% of patients in this analysis, this result may be attributed to an overlap between gastrointestinal events associated with pimitespib treatment and those associated with the progressive worsening of advanced GIST.

Ocular toxicity has been considered a class effect of HSP90 inhibitors based on findings that this group of medications induce retinal damage and cytotoxicity [28–30]; as such, ocular ADRs were of particular clinical interest in this analysis. In the phase 1 study of pimitespib in patients with advanced solid tumors, the incidence and severity of ocular AEs were lower with QD dosing for 5 consecutive days followed by 2 days’ rest per week (when compared with standard QD dosing) [17]; therefore, this regimen was adopted in the subsequent phase 2 and 3 studies of pimitespib in advanced GIST [18, 19]. As a result, the incidence of ocular ADRs in this pooled analysis was 22%, most ocular ADRs (92%) were grade 1 in severity, and no grade ≥ 3 events were observed. The onset of ocular ADRs after pimitespib initiation typically occurred later than gastrointestinal ADRs, but they also tended to recover earlier. Moreover, relatively few patients required pimitespib dose modifications in order to manage their ocular ADRs, and the outcome of these events was recovered/recovering for the majority of patients. Nevertheless, it remains important to conduct an ophthalmological examination if ocular ADRs occur during pimitespib treatment, and to take all appropriate measures to ensure that they are promptly resolved.

Despite being a pooled analysis, the main limitation of this study is its small sample of 119 Japanese clinical trial participants. Consequently, this may limit the generalizability of our findings to other countries and to the wider range of patients seen in routine clinical practice. A potential for selection bias may be applicable since all the data were from Japanese patients. It is worthy, however, to note that a previous report showed no ethnic differences in safety profiles of pimitespib between Japanese and Caucasian patients [17], suggesting that our findings may be generalizable to people of other ethnicities. Further evaluation is warranted; data from ongoing studies (such as NCT05245968, which includes non-Japanese populations in the European Union and USA), and real-world data studies will assist in this regard [31, 32]. Post-marketing surveillance of the safety and tolerability of pimitespib is ongoing, and will provide data on its safety in everyday clinical practice. Another possibility of selection bias may arise because eligibility criteria included only participants with an Eastern Cooperative Oncology Group Performance Status of 0 or 1.

In conclusion, this pooled safety analysis demonstrated the tolerability of pimitespib in Japanese patients with advanced GIST. Gastrointestinal ADRs that are common to HSP90 inhibitors were mostly mild or moderate in severity, and were manageable with treatment modifications and/or supportive therapies. Similarly, ocular ADRs with pimitespib – consistent with those associated with HSP90 inhibitors – were generally mild, and most were manageable with treatment modifications. In conjunction with efficacy data from these trials, our results further support the use of pimitespib for the treatment of patients with advanced GIST in Japan.

## Disclosure

YK reports consulting fees from Astellas Pharma and Taiho Pharmaceutical Co., Ltd.; grants or contracts from AstraZeneca, MSD, Ono Pharmaceutical, and Yakult Honsha; and honoraria or speaker fees from Astellas Pharma, Daiichi Sankyo, Eli Lilly, MSD, Nippon Kayaku, Ono Pharmaceutical, Taiho Pharmaceutical Co. Ltd., and Yakult Honsha. NY reports an employment/leadership position/advisory role for Eisai, Boehringer Ingelheim, CMIC, Chugai Pharmaceutical Co. Ltd., Merck, Healios, Mitsubishi Tanabe Pharma, Rakuten Medical, IQVIA, Noile-Immune Biotech, and Janssen; honoraria from Chugai Pharmaceutical Co. Ltd., Daiichi Sankyo, and Eisai; and research funding from Astellas, Chugai Pharmaceutical Co. Ltd., Eisai, Taiho Pharmaceutical Co. Ltd., Bristol Myers Squibb, Pfizer, Novartis, Eli Lilly, AbbVie, Daiichi Sankyo, Bayer, Boehringer Ingelheim, Kyowa Kirin, Takeda, Ono Pharmaceutical, Janssen Pharma, MSD, Merck, GSK, Chiome Bioscience, Otsuka Pharmaceutical, and Carna. YH, NA, and CS are employees of Taiho Pharmaceutical Co., Ltd. TD reports consulting fees from A2 Healthcare, Boehringer Ingelheim, Chugai Pharmaceutical Co. Ltd., Janssen Pharmaceuticals, Kaken Pharmaceutical Co. Ltd., Kyowa Kirin, Mitsubishi Tanabe Pharma, Noile-Immune Biotech, Oncolys BioPharma, Otsuka Pharmaceutical, PRA Health Sciences, Rakuten Medical, Shionogi, Sumitomo Pharma, and Takeda; grants or contracts from AbbVie, Amgen, Bayer, Boehringer Ingelheim, Bristol Myers Squibb, Chugai Pharmaceutical Co. Ltd., Daiichi Sankyo, GSK, Janssen Pharmaceuticals, Kyowa Kirin, MSD, Ono Pharmaceutical, Pfizer, PRA Health Sciences, RIN Institute, Shionogi, and Taiho Pharmaceutical Co. Ltd.; and honoraria or speaker fees from Daiichi Sankyo.
